# Antimicrobial, Antiproliferative and Proapoptotic Activities of Extract, Fractions and Isolated Compounds from the Stem of *Erythroxylum caatingae* Plowman

**DOI:** 10.3390/ijms13044124

**Published:** 2012-03-29

**Authors:** Jaciana S. Aguiar, Rosilma O. Araújo, Maria do Desterro Rodrigues, Kêsia X. F. R. Sena, André M. Batista, Maria M. P. Guerra, Steno L. Oliveira, Josean F. Tavares, Marcelo S. Silva, Silene C. Nascimento, Teresinha Gonçalves da Silva

**Affiliations:** 1 Antibiotics of Departament, Federal University of Pernambuco, 50670-901, Recife, PE, Brasil; E-Mails: jacianaaguiar@gmail.com (J.S.A.); rosilma23@hotmail.com (R.O.A.); mdrodrigues@yahoo.com.br (M.D.R.); kxfrs@bol.com.br (K.X.F.R.S.); silenen@yahoo.com.br (S.C.N.); 2 Andrology Laboratory, Veterinary Medicine Department, Rural Federal University of Pernambuco, 52171-900, Recife, PE, Brasil; E-Mails: mariannob@yahoo.com.br (A.M.B.); mpguerra@dmv.ufrpe.br (M.M.P.G.); 3 Laboratory of Pharmaceutical Technology, Department of Pharmaceutical Sciences, Federal University of Paraiba, Cx. Postal 5009, 58051-970, João Pessoa, Paraíba, Brasil; E-Mails: stenolacerda@yahoo.com.br (S.L.O.); joseanfechine@yahoo.com.br (J.F.T.); marcelosobral@pq.cnpq.br (M.S.S.)

**Keywords:** *Erythroxylum caatingae*, antimicrobial activity, cytotoxic activity, hemolytic activity, apoptosis

## Abstract

In the study, we have examined the antitumor and antimicrobial activities of the methanol extract, the fractions, a fraction of total alkaloids and two alkaloids isolated from the stem of *Erythroxylum caatingae* Plowman. All test fractions, except the hexane fractions, showed antimicrobial activity on gram-positive bacteria and fungi. The acetate: methanol (95:5), acetate, chloroform and hexane fractions show the highest cytotoxicity activity against the NCI-H292, HEp-2 and K562 cell lines using MTT. The absence of hemolysis in the erythrocytes of mice was observed in these fractions and 6β-Benzoyloxy-3α-(3,4,5- trimethoxybenzoyloxy) tropane (catuabine B). Staining with Annexin V-FITC and JC-1 was used to verify the mechanism of action of the compounds of *E. caatingae* that showed cytotoxicity less than 30 μg/mL in leukemic cells. After 48 h of incubation, we observed that the acetate: methanol (95:5), acetate, and chloroform fractions, as well as the catuabine B, increased in the number of cells in early apoptosis, from 53.0 to 74.8%. An analysis of the potential of the mitochondrial membrane by incorporation of JC-1 showed that most cells during incubation of the acetate: methanol (95:5) and acetate fractions (63.85 and 59.2%) were stained, suggesting the involvement of an intrinsic pathway of apoptosis.

## 1. Introduction

The research with medicinal plants aiming at the development of phytotherapeutic medicines and the promotion of the rational use of these products by the populations of developing countries such as Brazil, have a great importance not only in socio-economic aspects but also because they enable a greater knowledge of the culture of such people and a better utilization of the biodiversity of the respective countries [1]. Approximately 25% of the medicines prescribed in the industrialized countries originate from plants and about 120 compounds of natural origin, obtained from approximately 90 species of plants, are used in modern therapy. In Brazil, approximately 80,000 species of plants are described, offering a wide range of raw material for the discovery of new drugs [2,3].

It is well established that plants have always been useful sources of antitumor or cancer prevention compounds [4–6]. Approximately more than 60% of currently-used anticancer chemotherapeutic drugs are derived in one way or another from natural sources, including plants [7,8]. The search for anticancer agents from plant sources began in the 1950s with the discovery and development of the vinca alkaloids, vinblastine and vincristine, isolated from *Catharanthus roseus*, and etoposide and teniposide, the semisynthetic derivatives of epipodophyllotoxin, isolated from *Podophyllum peltatum* and *P. endoii* [7].

The indiscriminate use of antibiotics has led to antibiotic resistance in many pathogenic microorganisms and has led researchers to seek new antibiotics that are effective. Several alternatives have been suggested to solve this problem, such as the search for new antimicrobials in plant species [9]. Since the early 1980s, the number of drugs in development has decreased considerably, while the resistance of microorganisms, due to the constant use of antibiotics, has grown immensely. This is because series of new mechanisms of resistance are constantly being developed. The study of bacterial resistance is usually based on microorganisms of epidemiological significance, such as *Staphylococcus aureus*, *Escherichia coli*, *Pseudomonas aeruginosa* and fungi, which are responsible for different etiological processes in both immunocompetent and immunosuppressed patients [10].

The Erythroxylaceae family comprises approximately 250 species distributed across four genera, *Aneulophus* Benth, *Nectaropetalum* Engl., *Pinacopodium* Exell and Mendonça and *Erythroxylum* P. Browne [11,12]. The genus *Erythroxylum* consists of approximately 200 species, diversely distributed over South America, Africa and the island of Madagascar [13,14]. It has been used in many ethnomedical practices as an anti-inflammatory agent, an antibacterial agent and a tonic for its stimulant properties and as a powerful diuretic for liver, renal and vesicular afflictions. *Erythroxylum* has been used to treat venereal diseases, rheumatism, arthrosis, respiratory affections, amenorrhea and hemorrhages [15,16]. In Brazil, the family is represented by the genus *Erythroxylum*, with approximately 114 species, and there are 13 species registered in state of Paraíba [12].

The genus is characterized by the presence of tropane alkaloids, tannins, terpenes, flavonoids and phenylpropanoids [14,17]. Tropane alkaloids are an important class of natural products because of their analgesic, anesthetic, anticholinergic, antiemetic, antihypertensive, parasympatholytic, and other pharmacological properties [18].

The aim of the present study was to evaluate the antimicrobial and cytotoxic activities of the methanol extract fractions and two alkaloids isolated from the stem of *Erythroxylum caatingae* Plowman. For the evaluation of antimicrobial activity, the following fractions were tested against gram-positive and gram-negative bacteria and against fungi: the acetate phase (AcOEt), various ratios of acetate: methanol [AcOEt:MeOH (60:40); AcOEt:MeOH (80:20); AcOEt:MeOH (90:10) and AcOEt:MeOH (95:5)], chloroform (CHCl_3_) and hexane (C_6_H_6_). Antiproliferative activity was tested against tumor cell lines (NCI-H292, human lung mucoepidermoid carcinoma cells, K562, chronic myelocytic leukemia, and HEp-2, human larynx epidermoid carcinoma cells) and hemolytic activity was measured for the methanol extracts, its fractions and alkaloids. Another set of experiments was performed with K562, which was used to investigate the mechanisms involved in the antitumor activity of the fractions and the alkaloids of *E. caatingae*.

## 2. Results and Discussion

### 2.1. Antimicrobial Activity

The antimicrobial activities of methanol extract, fractions from the stem of *E. caatingae* Plowman were tested using the disc diffusion method at a concentration of 2000 μg/disc. The results are presented in Tables 1–3. All test fractions, except the hexane fractions, showed antimicrobial activity on gram-positive bacteria and fungi. The DMSO (control) did not produce inhibition haloes against the microorganisms studied, indicating that this solvent does not interfere with the antimicrobial activity results for the extract or fractions. The bacteria that were the most sensitive to the fractions tested were *Micrococcus luteus* and *Mycobacterium smegmatis*, with each showing inhibition zones of 13.5 ± 0.7 to 29.5 ± 0.7 mm and 14.0 ± 1.4 to 31.5 ± 1.4 mm, respectively. The AcOEt:MeOH (80:20), AcOEt:MeOH (90:10), AcOEt:MeOH (95:5) and the CHCl_3_ phases showed significant inhibition zones of 18.0 ± 0.0 to 19.0 ± 1.4 mm for *Candida albicans* (Table 1). Evaluation of the minimum inhibitory concentration of the extract and fractions of *E. caatingae* (zone of inhibition > 12 mm) showed significant inhibitory concentrations for *S. aureus*, *M. luteus*, *B. subtilis*, *M. smegmatis*, *E. faecalis* and *C. albicans*. However, the lowest values of the minimum inhibitory concentration (MIC) and minimum bacteriostatic concentration (MBC) against *M. luteus* went to the AcOEt:MeOH (95:5), AcOEt:MeOH (90:10) and CHCl_3_ fractions (Tables 1–3).

Plants are an important source of potentially useful structures for the development of new chemotherapeutic agents. The first step to identification of new chemotherapeutic agents is through trials of antibacterial activity *in vitro*. Many reports are available on the antiviral, antibacterial, antifungal, anthelmintic, antimolluskal and anti-inflammatory properties of plants. Some of these observations have proven helpful in the identification of the active principle compounds responsible for such activities and in the development of drugs for therapeutic use in humans [19].

There are a few reports of the antimicrobial activity of the genus *Erythroxylum*. Among them, we highlight the alkaline extract of the bark of *Erythroxylum catuaba* that has been shown to have a protective action against lethal infections of *E. coli* and *S. aureus* [20]. However, Rahman *et al*. (1998) [21] reported the absence of antibacterial activity against *S. aureus*, *E. coli*, *B. subtilis* and *P. aeruginosa* at the concentrations of 100 and 200 μg/mL and the presence of antifungal activity of the alcoholic extract of *Erythroxylum moonii* against *C. albicans*. In our study, the absence of any antimicrobial activity of *E. caatingae* against *S. aureus*, *P. aeruginosa* and *E. coli* was also verified, while the AcOEt:MeOH (80:20), AcOEt:MeOH (90:10), AcOEt:MeOH (95:5) and the CHCl_3_ fractions inhibited the growth of *C. albicans*. However, the lowest values of MIC and MBC went to the AcOEt:MeOH (90:10), AcOEt:MeOH (95:5) and chloroform fractions against *M. luteus.* It was observed from the data that the most active fractions were more polar, probably due to the presence of phenolic compounds such as flavonoids or tannins. According Haslan (1996) [22], the antimicrobial activity exhibited by tannins is explained by the ability of these compounds present in complexing with macromolecules, such as polysaccharides and proteins present in bacteria.

### 2.2. Cytotoxicity Activity

The cytotoxicity of the 11 samples, including the extract, fractions and two isolated compounds on the human tumor cell lines were evaluated after 72 h using MTT, and the results are presented in Table 4. The AcOEt:MeOH (95:5) (5), AcOEt (6), CHCl_3_ (7) and C_6_H_6_ (8) fractions were the most cytotoxic against the NCI-H292, HEp-2 and K562 cell lines. The most sensitive strains tested were the HEp-2 cell line, with an IC_50_ value equal to 8.25 ± 0.36 μg/mL for the CHCl_3_ fraction, and the K562 cell line, with an IC_50_ value equal to 13.10 ± 0.63 μg/mL, 9.86 ± 0.56 μg/mL and 11.21 ± 0.46 μg/mL for the AcOEt:MeOH (95:5), AcOEt and CHCl_3_ fractions, respectively. To verify whether the cytotoxicity observed was related to the injury of the cell plasma membrane, fractions 3–8 (Table 4) and the Catuabine B of *E. caatingae* were tested in the erythrocytes of mice (*Mus musculus)*. The absence of hemolysis was observed in all substances.

The cytotoxic activity of *Erytroxylum* in carcinoma and adenocarcinoma cells was first described by Silva *et al*. (2001) [15]. This study showed that the methanolic extract and alkaloids of tropanes of *Erytroxylum pervillei* inhibited the growth of KB-V1 cells (human cervix carcinoma multidrug-resistant cells) in the presence of vinblastine and were much less cytotoxic to KB-V1 cells in the absence of vinblastine or KB cells (oral squamous cell carcinoma). The alkaloids of the species pervillene B and C showed cytotoxic activity in adenocarcinomas of ovary (SKOV3) cells and in adenocarcinomas of multidrug-resistant ovary (SKVLB) cells incubated with adriamycin. The following year, alkaloids isolated from the stem of *Erytroxylum rotundifolium* were also found to be cytotoxic in KB-V1 cells [23].

Extracts of *Erytroxylum minutifolium* and *Erytroxylum confusum* produced a significant cytotoxic effect in rat hepatocytes and displayed hepatoprotective, anti-inflammatory and antimicrobial activities [24,25].

Among the few reports of cytotoxicity of the genus *Erythroxulum,* our group found an absence of cytotoxicity in the methanol extract of *E. caatingae* stems and in the catuabine B against human cancer cell lines (HEp-2, NCI-H292 and KB) of cytotoxicity within in the tested concentration range of 50.0–1.56 μg/mL [26]. However, this cytotoxicity test showed that the AcOEt: MeOH (95:5), AcOEt, CHCl_3_ and hexane fraction were the most active against the HEp-2, NCI-H292 and K562 cell lines. The extracts showed promising activity, with IC_50_ values ranging between 8.2–40.5 μg/mL. Most of these values were within the cutoff point of the National Cancer Institute criteria for cytotoxicity (IC_50_ < 30 μg/mL) in the screening of crude plant extracts, which indicates that these extracts are promising for further purification and study [27].

Although we observed cytotoxic activity, the *E. caatingae* extracts and fraction did not possess any activity against the mouse erythrocytes. These data suggest that the cytotoxic activity was not related to the lytic properties or the membrane instability induced by the extracts.

### 2.3. Study of the Mechanisms of Action of the Phases of Erythroxylum caatingae

#### 2.3.1. Annexin/PI Cell Death Assay

**Figure 1:**
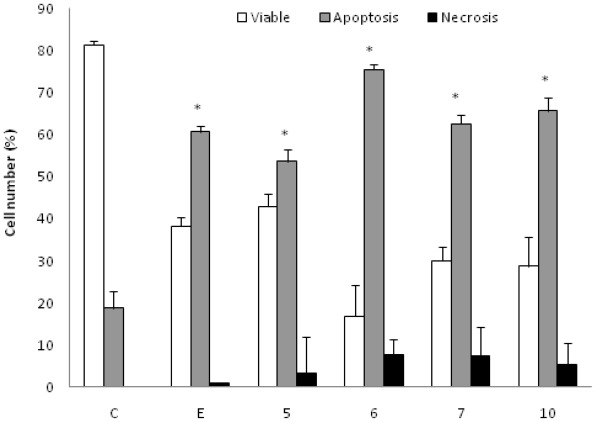
Effect of AcOEt:MeOH (95:5) (**5**), AcOEt (**6**), CHCl_3_ (**7**) and Catuabine B (**10**) in K562 cell population determined by fluorescence microscopy using Annexin V—FITC Kit, after 48 h incubation. The negative control (**C**) was the vehicle used for diluting the tested substances. Etoposide (**E**) was used as positive control. * *p* < 0.01 compared to control by ANOVA, followed by Newman-Keuls multiple comparison test. Data are presented as mean ± SD from three independent experiments.

**Figure 2:**
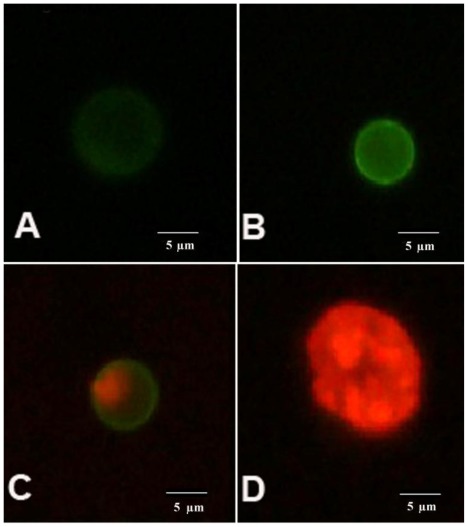
AnnV/PI staining of the cells. An image of K562 showing: (**A**) viable (AnnV^neg^/PI^neg^); (**B**) early apoptotic (AnnV^pos^/PI^neg^); (**C**) late apoptotic (AnnV^pos^/PI^pos^) and (**D**) necrotic (AnnV^neg^/PI^pos^) cell.

K562 cells incubated with the AcOEt:MeOH (95:5) (5), acetate (6) and CHCl_3_ fractions (7) and the catuabine B (10) were evaluated with the Annexin V-FITC kit by fluorescent microscopy to verify that cell death was induced by apoptosis through the externalization of phosphatidylserine. The doses chosen for the test were equal to the IC_50_ values. After 48 h of incubation, we observed that the AcOEt:MeOH (95:5) (13 μg/mL), AcOEt (9.8 μg/mL) and CHCl_3_ (11.2 μg/mL) fractions, as well as the catuabine B alkaloid (10 μg/mL), all increased the number of cells in early apoptosis (Ann V^pos^/PI^neg^) by 53.0%, 74.8%, 61.7% and 65.0%, respectively, when compared to the control. These substances exhibited values less than 1% of late apoptotic cells (AnnV^pos^/PI^pos^) and values less than 8% of cells in necrosis (AnnV^neg^/PI^pos^) (Figures 1 and 2).

Apoptosis is currently a subject of research interest, in part because tumor cells are susceptible to death by apoptosis in response to various drugs used in chemotherapy [28].

Apoptosis is regulated by two major pathways: the extrinsic (receptor mediated) and intrinsic (mitochondrial mediated) pathways. Due to the sensitivity of the intrinsic pathway, tumors arise more often through this pathway than through the extrinsic pathway [29]. The extrinsic pathway involves execution through cell surface death receptors, recruiting the Fas-associated death domain, which leads to the activation of caspase-8 [30]. The intrinsic pathway requires a loss/disruption of the mitochondrial membrane potential in the cells, which eventually may cause the initiation and activation of apoptotic cascades. The cascade triggers the release of cytochrome c and other apoptogenic molecules, such as Smac/DIABLO, from the mitochondria to the cytosol. Once in the cytosol, cytochrome c binds to Apaf-1 and recruits and activates caspase-9 in the apoptosome. Active caspase-9 cleaves and activates caspase-3, the caspase required to complete the induction of apoptosis [31]. Therefore, we sought to determine whether the phases that induced apoptosis were associated with the disruption of the mitochondrial membrane potential in K562 cells. As shown in Figure 2A, the treatment of K562 cells with the AcOEt:MeOH (95:5) (13 μg/mL), AcOEt (9.8 μg/mL) and CHCl_3_ fraction (11.2 μg/mL), as well as the catuabine B for 48 h resulted in an increase in the number of cells in early apoptosis by 53.0, 74.8, 61.6 and 65.0%, respectively. These data show that the main mechanism of cell death caused by the extracts is apoptosis, which suggests the involvement of the intrinsic pathway.

#### 2.3.2. Measurement of the Mitochondrial Membrane Potential

**Figure 3:**
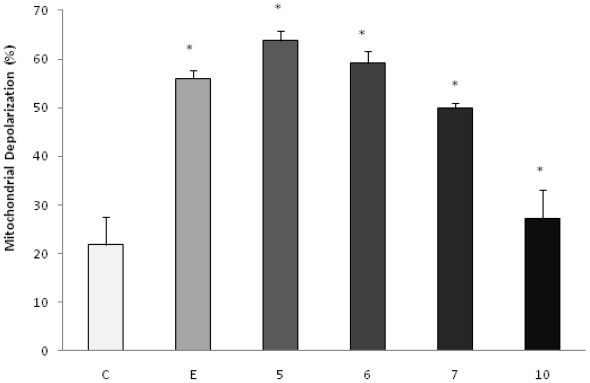
Effect of AcOEt:MeOH (95:5) (**5**), AcOEt (**6**), CHCl_3_ (**7**) and catuabine B (**10**) in K562 cell population determined by fluorescence microscopy using JC-1, after 48 h incubation. The negative control (**C**) was the vehicle used for diluting the tested substances. Etoposide (**E**) was used as positive control. * *p* < 0.01 compared to control by ANOVA, followed by Newman-Keuls multiple comparison test. Data are presented as mean ± SD from three independent experiments.

**Figure 4:**
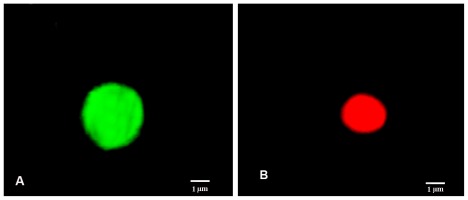
JC-1 staining of the cells. An image of K562 showing: (**A**) Green fluorescence emitted by cells is attributed to depolarized mitochondrial membrane; (**B**) No depolarized mitochondrial membrane emits red fluorescence.

To investigate the possible involvement of the mitochondrial pathway in apoptosis induced by the products under study, the analysis of the mitochondrial membrane potential by the incorporation of JC-1 was evaluated by fluorescence microscopy. The doses chosen for the test were equal to the IC_50_ values. After 48 h of incubation, we observed that the AcOEt:MeOH (95:5), AcOEt and CHCl3 fractions, as well as the catuabine B (10), all showed 63.8%, 59.2%, 50.0% and 27.2% apoptotic cells, respectively (Figures 3 and 4).

To confirm the involvement of the intrinsic pathway in cell death induced by treatment with the fractions and the alkaloids isolated from *E. caatingae,* an analysis of the mitochondrial membrane potential was made by the incorporation of JC-1. Cells treated with the AcOEt:MeOH (95:5), AcOEt, and CHCl_3_ fractions, as well as the catuabine B for 48 h, resulted in an increase in the number of green fluorescence-positive cells of 63.8, 59.2, 50.0, and 27%, respectively, thus, confirming the disruption of the mitochondrial membrane potential on treatment.

The intrinsic pathway requires the disruption of the mitochondrial membrane and the release of mitochondrial proteins, such as cytochrome c, which work together with the other two cytosolic protein factors, Apaf-1 (apoptotic protease activating factor-1) and procaspase-9, to promote the assembly of the caspase-activating complex (termed the apoptosome), which in return induces the activation of caspase-9 and initiates the apoptotic caspase cascade [32,33]. In our study, the AcOEt:MeOH (95:5), AcOEt, and CHCl_3_ fractions showed death by apoptosis, which mainly involved the mitochondrial pathway, especially with the acetate fraction.

## 3. Experimental Section

### 3.1. Chemicals

Phosphate buffered saline (PBS), penicillin-streptomycin liquid and DMEM (Dulbecco’s Modified Eagle’s Medium) were purchased from Gibco. MTT (3-(4,5-Dimethyl-2-thiazolyl)-2,5-diphenyl-2*H-*tetrazolium bromide) was purchased from Invitrogen. Eposide (Etoposide) was purchased from Blausiegel. Fetal bovine serum (FBS), glutamine, Triton X-100 and JC-1 (5,5′,6,6′-Tetrachloro-1,1′, 3,3′-tetraethylbenzimidazolcarbocyanine iodide) were purchased from Sigma-Aldrich Brazil. The Annexin V-FITC Apoptosis Detection Kit was purchased from Calbiochem. DMSO (Dimethyl sulfoxide), CaCl_2_, NaCl, and paraformaldehyde were purchased from Vetec. Mueller Hinton Agar and Sabouraud were purchased from Difco, and kanamycin and ketoconazole were purchased from Cecon.

### 3.2. Plant Material

The stems of *Erythroxylum caatingae* Plowman were collected in Picuí, Paraíba, Brazil. The botanical material was identified by Maria de Fátima Agra of the Laboratory of Pharmaceutical Technology, Federal University of Paraíba. A dried specimen was deposited in the herbarium of Lauro Pires Xavier (JPB) of the Federal University of Paraíba under the identification label AGRA 5666.

### 3.3. Extraction and Isolation

The crude methanol extract (500 g) was dissolved in water and defatted with C_6_H_6_. The defatted aqueous extract was acidified with 3% HCl with mechanical mixing and filtered through Celite, which yielded a residue, which was discarded, and an acidic solution. The acidic solution was extracted with CHCl_3_ (3 × 500 mL), which resulted in an acid chloroform phase and an aqueous phase that was neutralized to pH 7.0 with ammonium hydroxide. The aqueous phase, at pH 7.0, was extracted with CHCl_3_ to yield an aqueous phase and the basic chloroform phase (4.0 g), which was submitted to column chromatography (CC) using a silica gel as the stationary phase and CHCl_3_ and MeOH as the eluents, which were used either independently or in binary mixtures with increasing polarity. The result was 55 fractions of 100 mL. The 55 fractions were monitored by thin layer chromatography (TLC) and eluted with various solvent systems (CHCl_3_ and/or CHCl_3_-MeOH in order of increasing polarity) in chambers that were pre-saturated with NH_3_ vapor. They were then revealed with Dragendorff’s reagent and placed in 21 groups based on their Rf. Fraction 25, 45 and 46 were submitted to repeated recrystallization with acetone and ether to obtain alkaloids. The elucidation of the chemical structures was described by Oliveira *et al*. (2011) [26]. 6β-Benzoyloxy-3α-(3,4,5-trimethoxybenzoyloxy) tropane (Catuabine B) and 3α,6β-dibenzoyloxytropane were also isolated. The extract and fractions obtained were evaporated until dry. The following extracts were all tested: the methanol extract of the stem of *E. caatingae* (MEEC) and the AcOEt:MeOH (60:40), AcOEt:MeOH (80:20), AcOEt:MeOH (90:10), AcOEt:MeOH (95:5), AcOEt, CHCl_3_, and the hexane fractions, as well as the total alkaloids fraction.

### 3.4. Microorganisms

The microorganisms, which were from the Antibiotics Department collection of the Federal University of Pernambuco were as follows: *S. aureus*, UFPEDA 01; *Bacillus subtilis*, UFPEDA 16; *Enterococcus faecalis*, UFPEDA 138; *Micrococcus luteus*, UFPEDA 06; *Escherichia coli*, UFPEDA 224; *P. aeruginosa*, UFPEDA 39; *Serratia marcescens*, UFPEDA 398; *Mycobacterium smegmatis*, UFPEDA 71; and *Candida albicans*, UFPEDA 1007, and these were used in the antimicrobial activity assays.

### 3.5. Cell Lines and Cell Culture

The cell lines used for the *in vitro* cytotoxicity tests were K562 (human chronic myelocytic leukemia), NCI-H292 (human lung mucoepidermoid carcinoma cells) and HEp-2 (human larynx epidermoid carcinoma cells), and these were obtained from the Adolph Lutz Institute (São Paulo, Brazil). The cells were maintained in DMEM supplemented with 10% fetal bovine serum, 2 mM glutamine, 100 U/mL penicillin, and 100 μg/mL streptomycin at 37 °C with 5% CO_2_.

### 3.6. Animals

Three Swiss mice (female, 25–30 g), obtained from the central animal house of the Universidade Federal de Pernambuco, Brazil, were used. The animals were housed in cages with free access to food and water. All animals were kept under a 12 h light:12 h dark cycle (lights on at 6:00 a.m.). The animals were treated according to the ethical principles of animal experimentation of the Brazilian College of Animal Experimentation (COBEA), Brazil. The Animal Studies Committee of the Universidade Federal do Pernambuco approved the experimental protocols (Number 23076.012173/2007-77).

### 3.7. Antimicrobial Activity

Antimicrobial activity was verified *in vitro* by the method of diffusion in paper record [34]. The microorganisms were standardized with an optical density of McFarland 0.5 in physiological solution [35], which corresponds to a concentration of approximately 10^7^ UFC/mL for yeast and filamentous fungi and 10^8^ UFC/mL for bacteria. On the inoculated medium, Mueller Hinton Agar (*S. aureus*, *B. subtilis*, *M. luteus*, *E. coli*, *P. aeruginosa* and *S. marcescens*), glucose extracts of yeast (*E. faecalis*, *M. smegmatis*) and Sabouraud (*C. albicans*), and discs of barren paper (6 mm) were absorbed with 10 μL of the solution 200,000 μg/mL of the methanol extract from the stem of *E. caatingae* and its fractions and were placed on the agar. After placing the discs, the plates were incubated for 24 h and 48 h at 30 °C and 35 °C, respectively. The antibiotics kanamycin and ketoconazole were used as standards at 30 μg/disc and 300 μg/disc, respectively.

The Minimum Inhibitory Concentration (MIC) and Minimum Bacteriostatic Concentration (MBC) were applied to the substances that showed halos larger than 12 mm by serial dilutions in half-solids [36]. Aliquots of different volumes (0.03–1.0 mL) of the solutions at 20.000 μg/mL were placed in Petri dishes and homogenized with 10 mL of the appropriate culture medium. The microorganisms were streaked on the surface of the medium, and the plates were incubated at 35 °C and 30 °C for 24 h and 48 h, respectively. For values over 1000 μg/mL, the extract was considered inactive [37].

### 3.8. Cell Viability Assay (MTT Assay)

The cytotoxicity of the methanol extract of the stems of *E. caatingae* and its fractions were tested against the K562, NCI-H292 and HEp-2 tumor cell lines. For these experiments, the cells were plated in 96-well plates (10^5^ cells/mL for adherent cells or 0.3 × 10^6^ cells/mL for suspended cells). After 24 h, the extracts, fractions (6.25–50 μg/mL) and alkaloids (1.25–10 μg/mL) dissolved in DMSO were added to each well and incubated for 72 h. Control groups received DMSO (0.2%) in DMEN. Etoposide (1.25–20 μg/mL) was used as a positive control. The growth of the tumor cells was quantified by the ability of the living cells to reduce yellow tetrazolium MTT (3-(4,5-dimethylthiazolyl-2)-2,5- diphenyltetrazolium bromide) to a blue formazan product [38,39]. At the end of the 72 h incubation period, MTT (5.0 mg/mL) was added to the plate. After three hours for the suspended cells or two hours for the adherent cells, the formazan product from the reduction of MTT was dissolved in DMSO, and the absorbance was measured using a multi-plate reader. The effect of the drug was quantified as the percentage of control absorbance the reduced dye at 450 nm (Multi-plate Reader Thermoplate).

### 3.9. Hemolytic Assay

The hemolytic assay was performed in 96-well plates, following the method previously described by Costa-Lotufo *et al*. (2005) [40]. Each well received 100 μL of a 0.85% NaCl solution containing 10 mM CaCl_2_. The first well served as the negative control and only contained the vehicle (10% DMSO). The second well contained 100 μL of the test substance that was diluted 1:2. The extracts were tested at concentrations ranging from 15.62 to 2000 μg/mL. The serial dilution continued until the 11th well. The last well received 20 μL of 0.1% Triton X-100 (in 0.85% saline) to obtain 100% hemolysis (positive control). Each well received 100 μL of a 2% suspension of mouse erythrocytes in 0.85% saline containing 10 mM CaCl_2_. After incubation at room temperature for 30 min and centrifugation, the supernatant was removed, and the released hemoglobin was measured by spectroscopic absorbance at 450 nm. Extracts with an EC_50_ value lower than 200 μg/mL were considered to be active.

### 3.10. Analysis of the Mechanisms Involved in the Cytotoxic Activity

The following experiments were performed to elucidate the mechanisms involved in the cytotoxic activity of the fractions and alkaloids on K562 cells after 48 h of incubation.

#### 3.10.1. Annexin/PI Cell Death Assay

Cellular apoptosis was stained with Annexin V and propidium iodide (PI) using an Annexin V—FITC kit (Calbiochem) following the protocol provided by the manufacturer. The suspension of K562 cells (0.3 × 10^6^ cells/mL) was seeded into 96-well plates. The plates were incubated at 37 °C with 5% CO_2_ for 24 h. After this period, the fractions and alkaloids that presented cytotoxicity were added at the concentration that was equal to the IC_50_ value. After 48 h of treatment, the cells were stained with Annexin V and PI following the manufacturer’s recommended protocol and were analyzed by an epifluorescence microscope (Carl Zeiss, Göttingen, Germany) at 1000× magnification under an oil immersion with an emission filter of LP 515 nm and excitation filter of BP 450–490 nm. A minimum of 200 cells were counted in each sample.

#### 3.10.2. Measurement of the Mitochondrial

Membrane Potential Mitochondrial depolarization was evaluated by the incorporation of JC-1. This assay was performed as previously described, with some modifications [41,42]. The JC-1 probe is freely permeable to cells and undergoes reversible transformation from a monomer to an aggregate form (J_agg_). The suspension of K562 cells (0.3 × 10^6^ cells/mL) was seeded into 96-well plates. The plates were incubated at 37 °C and 5% CO_2_ for 24 h. The fractions that presented cytotoxicity were added at a final concentration equal to the IC_50_ value. After 48 h of treatment, 50 μL was collected, incubated with JC-1 (0.15 mM in DMSO) for 10 min in the dark and then washed twice with PBS. The cells were fixed with 4% paraformaldehyde, mounted on a glass slide and observed under an epifluorescence microscope (Carl Zeiss, Göttingen, Germany) with 1000× magnification under an oil immersion with an emission filter of LP 515 nm and an excitation filter of BP 450–490 nm. A minimum of 200 cells were counted in each sample. The cells stained in red were classified as having a high potential mitochondrial membrane, while those stained in green were classified as having a low potential membrane.

### 3.11. Statistical Analysis

Data are presented as the mean ± S.D. The IC_50_ and EC_50_ values and their 95% confidence intervals were obtained by nonlinear regression using SigmaPlot 11.0 (version 11.0.1; Systat Software Inc.: Chicago, IL, USA, 2011). The differences between the experimental groups were compared by one-way analysis of variance (ANOVA), followed by Newman-Keuls test; the significance level was set at 1%.

## 4. Conclusions

According to the results of investigations, methanol extract of the stem of *Erythroxylum caatingae* and all tested fractions, except the hexane fraction, showed antimicrobial activity on gram-positive bacteria and fungi. The absence of hemolysis in the erythrocytes of mice was observed in these fractions and 6β-Benzoyloxy-3α-(3,4,5-trimethoxybenzoyloxy) tropane (catuabine B). We observed that the acetate:methanol (95:5), acetate, and chloroform fractions, as well as the catuabine B induced apoptosis in K562 cells. An analysis of the potential of the mitochondrial membrane by incorporation of JC-1 showed that most cells during incubation of the acetate:methanol (95:5) and acetate phases were stained, suggesting the involvement of an intrinsic pathway of apoptosis.

## Figures and Tables

**Table 1:** Antimicrobial activity of the methanol extract of the stem of *Erythroxylum caatingae* and of its fractions (2000 μg/disc).

	Zone of Inhibition (mm)									
	
Microorganisms	1	2	3	4	5	6	7	8	Kanamicin (30 μg/disc)	ketoconazole (300 μg/disc)
***S. aureus***	10.5 ± 0.7	10.0 ± 0.0	11.0 ± 0.7	12.0 ± 0.0	12.0 ± 0.0	0.0	10.5 ± 0.7	0.0	28	-
***M. luteus***	21.5 ± 2.1	26.0 ± 1.4	28.5 ± 0.7	28.5 ± 0.7	29.5 ± 0.7	13.5 ± 0.7	26.0 ± 0.0	0.0	34	-
***B. subitilis***	10.0 ± 0.0	10.0 ± 0.0	13.5 ± 0.7	13.5 ± 0.7	13.0 ± 0.7	0.0	12.0 ± 0.0	0.0	29	-
***M. smegmatis***	14.0 ± 1.4	22.5 ± 0.7	30.0 ± 0.0	31.5 ± 1.4	30.0 ± 0.0	15.0 ± 0.0	15.0 ± 0.0	0.0	40	-
***E. faecalis***	0.0	0.0	11.5 ± 0.7	11.5 ± 0.7	12.5 ± 0.7	0.0	17.0 ± 0.0	0.0	13	-
***E. coli***	0.0	0.0	0.0	0.0	0.0	0.0	0.0	0.0	15	-
***S. marcescens***	0.0	0.0	0.0	0.0	0.0	0.0	0.0	0.0	15	-
***P. aeruginosa***	0.0	0.0	0.0	0.0	0.0	0.0	0.0	0.0	20	-
***C. albicans***	0.0	0.0	19.0 ± 1.4	19.0 ± 1.4	18.0 ± 1.4	0.0	18.0 ± 0.0	0.0	-	24

1: Methanol extract of stem of *E. caatingae* (MEEC); 2: AcOEt:MeOH (60:40); 3: AcOEt:MeOH (80:20); 4: AcOEt:MeOH (90:10); 5: AcOEt:MeOH (95:5); 6: AcOEt; 7: CHCl_3_; 8: Hexane fraction. Data are presented as the mean ± SD of two independent experiments. Each experiment was done in triplicate.

**Table 2:** Evaluates the minimum inhibitory concentration of the methanol extract of the stem of *Erythroxylum caatingae* and of its fractions.

Microorganisms	Minimum Inhibitory Concentration (μg/mL)						

	1	2	3	4	5	6	7
***S. aureus***	n.t.	n.t.	n.t.	250	250	n.t.	n.t.
***M. luteus***	250	250	n.t.	<31.25	<31.25	250	<31.25
***B. subtilis***	n.t.	n.t.	n.t.	250	250	n.t.	125
***M. smegmatis***	500	500	n.t.	125	125	1000	125
***E. faecalis***	n.t.	n.t.	n.t.	n.t.	500	n.t.	500
***C. albicans***	n.t.	n.t.	n.t.	250	125	n.t.	125

1: Methanol extract of stem of *E. caatingae* (MEEC); 2: AcOEt:MeOH (60:40); 3: AcOEt:MeOH (80:20); 4: AcOEt:MeOH (90:10); 5: AcOEt:MeOH (95:5); 6: AcOEt; 7: CHCl_3_; n.t.: not tested. Each experiment was done in triplicate.

**Table 3:** Evaluates the minimum bacteriostatic concentration of the methanol extract of the stem of *Erythroxylum caatingae* and of its fractions.

Microorganisms	Minimum Bacteriostatic Concentration (μg/mL)						

	1	2	3	4	5	6	7
***S. aureus***	n.t.	n.t.	n.t.	1000	500	n.t.	n.t.
***M. luteus***	250	500	n.t.	<31.25	<31.25	>2000	<31.25
***B. subtilis***	n.t.	n.t.	n.t.	1000	500	n.t.	250
***M. smegmatis***	1000	1000	n.t.	500	250	1,000	250
***E. faecalis***	n.t.	n.t.	n.t.	n.t.	1000	n.t.	1000
***C. albicans***	n.t.	n.t.	n.t.	500	500	n.t.	250

1: Methanol extract of stem of *E. caatingae* (MEEC); 2: AcOEt:MeOH (60:40); 3: AcOEt:MeOH (80:20); 4: AcOEt:MeOH (90:10); 5: AcOEt:MeOH (95:5); 6: AcOEt; 7: CHCl_3_; n.t.: not tested. Each experiment was done in triplicate.

**Table 4:** Cytotoxic and hemolytic activity de *Erytroxylum caatingae*.

Products/Extracts	Cell Line, IC_50_ (μg/mL)			EC_50_ (μg/mL)

	HEp-2	NCI-H292	K562	
**1**	>50	>50	>50	n.t.
**2**	>50	>50	>50	n.t.
**3**	>50	25.76 ± 1.71	>50	>2000
**4**	>50	25.50 ± 1.08	>50	>2000
**5**	40.59 ± 0.72	28.19 ± 2.09	13.10 ± 0.63	>2000
**6**	34.12 ± 1.01	20.37 ± 0.75	9.86 ± 0.56	403.24 ± 9.64
**7**	8.25 ± 0.36	34.39 ± 1.71	11.21 ± 0.46	1036.52 ± 35.17
**8**	31.42 ± 1.01	15.79 ± 1.01	33.58 ± 1.33	510.00 ± 8.60
**9**	>50	>50	>50	n.t.
**10**	>50	>50	9.36 ± 0.77	>250
**11**	>50	>50	>50	n.t.
**Etoposide**	6.10 ± 0.19	2.75 ± 0.10	4.48 ± 0.23	n.t.

1: Methanol extract of stem of *E. caatingae* (MEEC); 2: AcOEt:MeOH (40:60); 3: AcOEt:MeOH (80:20); 4: AcOEt:MeOH (90:10); 5: AcOEt:MeOH (95:5); 6: acetate fraction (AcOEt); 7: chloroform fraction (CHCl_3_); 8: hexane fraction (C_6_H_6_); 9: Fraction total of alkaloids; 10: catuabine B; 11: 3α,6β-dibenzoyloxytropane; n.t.: not tested. The IC_50_ and EC_50_ and its 95% confidence interval (CI 95%) were obtained by non-linear regression. Data are presented as mean ± SD of two independent experiments. Each experiment was done in triplicate.
